# Quantitative assessment of four‐dimensional computed tomography image acquisition quality

**DOI:** 10.1120/jacmp.v8i3.2362

**Published:** 2007-06-29

**Authors:** George Starkschall, Neil Desai, Peter Balter, Karl Prado, Dershan Luo, Dianna Cody, Tinsu Pan

**Affiliations:** ^1^ Departments of Radiation Physics The University of Texas M.D. Anderson Cancer Center Houston Texas U.S.A.; ^2^ Departments of Imaging Physics The University of Texas M.D. Anderson Cancer Center Houston Texas U.S.A.

**Keywords:** Computed tomography, 4D imaging, image assessment, organ motion

## Abstract

The purpose of the present work was to describe the development and validation of a series of tests to assess the quality of four‐dimensional (4D) computed‐tomography (CT) imaging as it is applied to radiation treatment planning. Using a commercial respiratory motion phantom and a programmable moving platform with a CT phantom, we acquired 4D CT datasets on two commercial multislice helical CT scanners that use different approaches to 4D CT image reconstruction. Datasets were obtained as the platform moved in various patterns designed to simulate breathing. Known inserts in the phantom were contoured, and statistics were generated to evaluate properties important to radiation therapy—namely, accuracy of phase‐binning, shape, volume, and CT number. Phase‐binning accuracy varied by as much as 5% for a 4D procedure in which images were reconstructed and then binned, but exhibited no variation for a 4D procedure in which projections were binned before reconstruction. The magnitude of geometric distortion was found to be small for both approaches, as was the magnitude of volume error. Partial‐volume effects in the direction perpendicular to the transverse planes of reconstruction affected volume accuracy, however. Computed tomography numbers were reproduced accurately, but 4D images exhibited more variation in CT number than static CT images did. Characterization of such properties can be used to better understand and optimize the various parameters that affect 4D CT image quality.

PACS numbers: 87.53.‐j, 87.59.Fm

## I. INTRODUCTION

To image the effects of motion using computed tomography (CT), techniques have been developed that take advantage of periodic or near‐periodic motion. These techniques involve the acquisition of projection information over small regions of the patient at various phases in the motion cycle, followed by the combination of information at specified phases over multiple motion cycles to generate a series of static images. This technique, known as four‐dimensional (4D) CT imaging, replaces time dependence with phase dependence, and is increasingly being used to image respiratory‐induced motion in the thorax and abdomen.

Two major techniques have been developed to acquire 4D CT images.

In one approach, a multislice helical CT scanner is operated in cine mode.^(1)^ Images are acquired at a specified table position for a period of time equal to at least one respiratory cycle plus one gantry rotation. The table is typically indexed a distance equal to the X‐ray beam width, and another set of images is acquired. This process is continued until the entire volume is scanned. The acquisition times of the transverse CT images obtained (typically 1500 – 3000 images) are correlated with information obtained from a respiratory monitor. Times corresponding with various phases in the respiratory cycle are identified, and transverse CT images acquired at or close to phase‐specific times are binned, resulting in the generation of multiple three‐dimensional (3D) datasets, one for each phase. We here refer to that technique as “image binning” (IB).

In another approach, the CT scanner is operated in helical mode at very low pitch.^(^ ^2^ ^,^ ^3^ ^)^ Projections are acquired, and the acquisition times of the projections are correlated with phase information from the respiratory monitor. Projections acquired in the vicinity of phase‐specific times are binned. These binned projections are reconstructed, resulting in multiple image sets, one for each phase. We here refer to that technique as “projection binning” (PB).

The goal of both techniques is to replace a time‐dependent CT image dataset with a respiratory phase–dependent CT image dataset. Inaccuracies in the 4D CT images acquired using either of these two methodologies may be the result of several factors.

**Figure 1:**
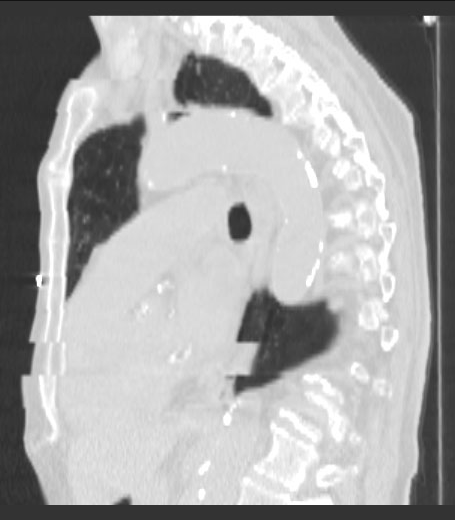
Sagittal view extracted from a four‐dimensional reconstruction illustrating an artifact caused by an irregular respiratory cycle. Note, in particular, the displacement approximately two thirds of the superior–inferior distance.

One factor is irregularities in the respiratory cycle. If the respiratory cycle is irregular, the relationship between the position of the patient and the phase location may be different in each respiratory cycle. These differences may cause positional artifacts such as that illustrated in Fig. 1. Note the severe displacement at approximately two thirds of the superior–inferior distance, caused by an irregular respiratory cycle.

Another source of inaccuracy in 4D CT imaging is patient motion. The reconstruction assumes that the patient is motionless during acquisition of a single set of CT images. In reality, the gantry of the CT scanner rotates at a finite speed, with a gantry rotation time that may be a short as approximately 0.4 sec. Consequently, an image acquired over a single gantry rotation may display motion artifacts.

The purpose of the present work was to describe the development and validation of a set of metrics used in assessing the quality of 4D CT images, particularly with regard to the use of these images for radiation treatment planning. From this viewpoint, the major imaging parameters to be assessed are those that might affect the eventual outcome of a radiation treatment plan, such as accuracy of phase‐binning, accuracy of volumes of regions of interest, accuracy of geometries of regions of interest, and accuracy of CT number.

Two different methods of CT image acquisition are demonstrated in this work; however, it is not a goal of the present work to compare these methods. Optimization of image acquisition and reconstruction parameters was not performed.

## II. MATERIALS AND METHODS

### A. Phase‐binning phantom

**Figure 2:**
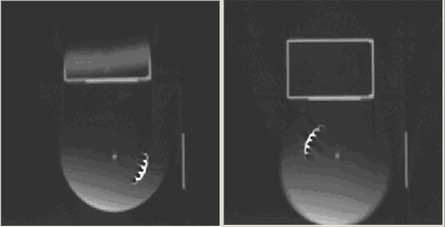
Transverse computed tomography images of the Respiratory Position Management phantom (Varian Medical Systems, Palo Alto, CA) reconstructed at 0% and 50% phase. The image of one ball bearing (BB) is in the center of the wheel; the blurred image of the second BB is approximately two thirds of the distance from the center to the edge of the wheel.

The first parameter assessed in this study was the accuracy of phase binning. To make that assessment, we used a motion phantom supplied by the vendor of the respiratory monitoring system [Respiratory Position Management (RPM): Varian Medical Systems, Palo Alto CA]. The phantom consists of a slightly irregular wheel that rotates approximately 9 times per minute. The reflective box from the respiratory monitoring system is placed on a platform against the wheel and moves vertically as the wheel rotates. This phantom is normally used as a tool to test the respiratory monitoring system. We modified the phantom slightly, placing two ball bearings (BBs) on the rotating wheel: one at the axis of rotation of the wheel, and a second several centimeters away from the axis of rotation. By determining the relative positions of the images of the two BBs on a CT dataset, we were able to determine the phase of the dataset, and we compared it with the desired phase. Fig. 2 illustrates transverse CT images of the motion phantom reconstructed at the 0% and 50% phases. The effect of a finite image acquisition time is evident in the blurring of the image of one BB. As a consequence of this blurring, we used the center BB position for phase determination.

### B. Motion platform and phantom

A platform capable of simulating the gross anatomic superior–inferior motion attributable to internal motion of the thoracic region has been described previously.^(4)^ The platform and base are made of clear polycarbonate plastic, one half inch in thickness, and the bearings are oriented to restrict the platform to displace a maximum distance of approximately 5 cm along a straight line. A stepper motor attached to a BiSlide (Velmex, Bloomfield, NY) drives the platform along a linear trajectory. The use of a BiSlide reduces the amount of inertial torque that a heavy load can impress directly upon the motor.

**Figure 3:**
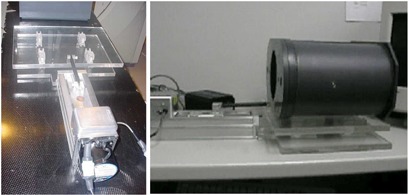
The Radiological Physics Center (RPC) platform, which allows for a component of motion in the transverse plane.

The spatial resolution of CT images along the patient axis (z direction) is the poorest, limited by our selection of a slice thickness of 2.5 – 3.0 cm, as compared with the submillimeter spatial resolution of images in the transverse plane. The lesser spatial resolution in the longitudinal direction proved problematic during our attempts to assess image distortion in the direction of motion. Consequently, the 4D CT image datasets were acquired using a platform developed for the Radiological Physics Center (RPC) that had a component of motion in the transverse plane.^(5)^ Fig. 3 illustrates the platform, which was programmed to move with various periodic trajectories that simulate a patient's respiratory cycle, including simulation of both regular and irregular breathing.

**Figure 4:**
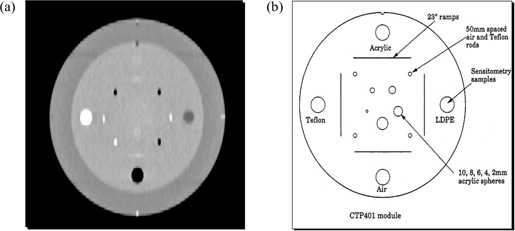
(a) Coronal reconstruction of the computed tomography image of the Catphan phantom (The Phantom Laboratory, Greenwich, NY). The reconstruction is somewhat elongated because of the motion of the platform. (b) Diagram of the cross‐section of the phantom. LDPE=low‐density polyethylene.

The 4D CT image datasets were acquired from a CT phantom (Catphan 500: The Phantom Laboratory, Greenwich, NY) mounted on the platform. The phantom is cylindrically shaped and includes several small cylindrical inserts of known composition. Fig. 4(a) shows an axial image of the phantom, and Fig. 4(b) illustrates the manufacturer's description of the inserts of interest. The phantom was placed on the RPC platform, long axis parallel to the CT table.

The phantom and moving platform were scanned using two commercial multislice helical CT scanners representing the two different approaches to 4D CT image reconstruction earlier described. One CT scanner represented the IB approach (Discovery ST PET/CT: General Electric HealthCare, Waukesha, WI); the other represented the PB approach (MX8000‐IDT: Philips Medical Systems, Cleveland, OH). The commercial respiratory monitoring system (RPM), which we currently use at our institution for 4D CT image acquisition, was used to monitor the motion of the platform. The system monitors respiration by using external fiducials to detect abdominal motion.

### C. Parameters to be assessed

Because our study was designed to evaluate the quality of 4D CT imaging with respect to radiation treatment planning, the specific parameters evaluated were those relevant to the radiation treatment planning process. In particular, we assessed the accuracy of the phase binning and of the geometries, volumes, and CT numbers of regions of interest in the phantom.

### D. Acquisition of 4D CT datasets

To assess the accuracy of phase binning, we obtained 4D CT image datasets of the RPM phantom. We used standard 4D thoracic image acquisition protocols to acquire image datasets of the CT phantom. For the Philips CT scanner (PB approach), we used 120 kVp, 400 mA per slice, a collimator setting of 16×1.5 mm, and 3‐mm slice reconstruction. For the GE scanner (IB approach), we used 120 kVp, 100 mA, a collimator setting of 8×2.5 mm, and 2.5‐mm slice reconstruction.

On the Philips CT scanner, a region approximately 15 cm in length was scanned with a pitch of 0.04 and a gantry rotation time of 0.4 s. Typical image acquisition times are in the vicinity 60 – 80 s. Tags were placed in the sinogram file at times corresponding to 0% phase as determined by the respiratory monitoring system. These tags corresponded to end inspiration.

Initially, we identified phases to be binned at nominal 10% intervals. The accuracy of the phase binning was assessed by determining the orientation of the line connecting the image of the BB at the axis of rotation of the phantom on the CT image dataset with the center of the image of the BB located away from the axis of rotation and by comparing the orientation of that line to the line on the 0% phase image dataset.

We found that the phases obtained from these nominal bins were not accurate. Correspondence with the vendor indicated that the nominal phases were a vestige from a previous version of the software, which had been developed for cardiac gating. With advice from the vendor, plus a small amount of experimentation, we obtained a set of nominal phase values that would provide us with accurate 10% phase intervals. Table 1 identifies these nominal phase intervals. However, it should be noted that in later software releases, the vendor corrected this issue, and phase values were determined to be accurate.

**Table 1:** Nominal phase values required yielding true phases in the Philips CT scanner

Nominal phase (%)	Actual phase (%)
0.0	0
20.7	10
33.2	20
42.0	30
50.3	40
58.3	50
66.0	60
74.0	70
81.7	80
90.4	90

Similar image acquisition was undertaken using the GE CT scanner with the RPM phantom. The GE scanner automatically bins images at 10% phase intervals and selects those images that are closest to the desired phases at the 10% phase intervals.

To assess image distortion and CT number accuracy, 4D CT images were acquired of the Catphan phantom using the RPC platform moving in these patterns:

- Periodic sinusoidal motion of 2‐cm amplitude at 15 cycles per minute, corresponding to a respiratory cycle of 4 s
- Periodic sinusoidal motion of 2‐cm amplitude at 20 cycles per minute, corresponding to a respiratory cycle of 3 s
- Almost‐sinusoidal motion with amplitude varying between 0.70 cm and 1.10 cm, and a period varying between 15 cycles and 20 cycles per minute

The amplitudes and frequencies of the “respiration” were selected to represent typical patient breathing patterns. Most motion associated with the respiratory cycle requires less than 2 cm displacement, and so the maximum range of motion was selected to be 2 cm. For the IB approach, typical cine duration was in the range 4 – 5 s, and time between images was in the range 0.2 – 0.3 s. For the PB approach, typical pitch was 0.08, with a gantry rotation time of 0.4 s. In all cases, the entire phantom was scanned.

Using the appropriate binning methodology, datasets were binned into phases at 10% intervals. To assess the geometric accuracy of the reconstruction, we measured the dimensions of the inserts in the x and y directions in a transverse plane, comparing the shape of the reconstructed images of known inserts with the shapes of the inserts on a static scan. To assess the accuracy of the CT numbers, we compared the mean CT numbers and standard deviations of the CT numbers of the inserts obtained on static scans with those extracted from 4D reconstructed scans.

### F. Analysis of 4D CT datasets

We segmented three of the cylindrical inserts [Teflon, low‐density polyethylene (LDPE), air] in the Catphan phantom using the threshold‐based segmentation algorithm provided on the treatment planning system. Threshold CT voxel values for the automatic segmentation were 1250 – 4096 for Teflon, 900 – 1000 for LDPE, and 0 – 900 for air. It should be noted that CT voxel values are expressed in the treatment planning system as 12‐bit unsigned integers ranging from 0 to 4095. Software tools in the treatment planning system were used to calculate the volumes of the inserts based on the segmentation.

To assess geometric distortion, the x and y dimensions of the inserts were transferred from the treatment planning system to a spreadsheet (Excel 2000: Microsoft Corporation, Redmond, WA). Because the inserts were cylindrical, deviations from circles in transverse planes served as a measure of distortion. The metric used was the ratio of the y dimension to the x dimension.

To assess consistency of CT voxel values, a circle of diameter 8 mm was drawn in a single transverse plane (2.5 – 3.0 mm) near the middle of each of the three phantom inserts. A software drawing tool generated a circle of 8 mm diameter. Software tools in the treatment planning system determined the maximum, minimum, mean, and standard deviation of the CT voxel values inside the circle, expressed, again, as 12‐bit unsigned integers.

## III. RESULTS

### A. Accuracy of phase binning

The data in Table 2 illustrate the accuracy in phase binning of the PB and IB approaches as measured by the RPM phantom. The PB approach gives very accurate phase binning, because projections are binned at precisely the phases input into the reconstruction software. With the IB approach, images are acquired at specific time intervals, and the images closest to the desired phase are selected for binning. Consequently, some phase error might result. This phase error can be reduced by reducing the time interval between image acquisitions to the temporal resolution of the CT scanner.

**Table 2:** Nominal and actual phase bins for a four‐dimensional computed tomography image of the Respiratory Position Management^a^ phantom as acquired using both binning approaches, after recalibration of the projection binning (PB) approach as indicated in Table 1

Nominal (%)	Actual PB (%)	Actual IB (%)
0	0	0
10	10	10
20	20	21
30	30	30
40	40	41
50	50	50
60	60	60
70	70	75
80	80	84
90	90	91

a Varian Medical Systems, Palo Alto, CA.

IB=image binning.

### B. Accuracy of geometric reproduction

Tables 3 – 5 show the results of the geometric reproduction of each of the three CT phantom inserts for the two reconstruction approach studies. The tables show the differences in elongation and volume of the cylindrical target inserts by comparing each phase of the 4D CT image dataset with a static dataset. Elongations occurred in the direction that corresponded to the vertical motion of the Catphan phantom. In general, elongations represented approximately 6% variation from the static images. However, part of the variance may be attributable to uncertainties in the outlining of the phantom inserts. The inserts were contoured using a CT thresholding algorithm with a threshold CT value approximately halfway between the CT voxel value of the insert and that of the surrounding phantom. Given the nature of thresholding, the estimated uncertainty in outlining a region of interest is at least 1 pixel (0.08 cm) based on a field of view of 40 cm. For these cylindrical target objects, with a typical dimension of 1.36 cm, the fractional uncertainty is 0.08/1.36=0.06. Adding uncertainties in the two directions in quadrature gives an uncertainty in elongation of approximately 0.08 (8%). Most calculated elongations were within 8% of the static image. Occasionally, elongation differences greater than 8% occurred—typically in the vicinity of the 20% or 30% phase, which corresponds with the greatest translational speed of the platform.

**Table 3:** Comparison of the distortion and volume of the image of the Teflon insert in each of 10 phases of a four‐dimensional image dataset with the image of the insert in a static computed tomography dataset

	Acquisition technique: 15 BPM, image binning				
	Dimension (cm)				
Phase (%)	AP	LR	Elongation^a^ (cm^3^)	Volume	Volume ratio^b^
Static	1.368	1.360	0.994	3.13	1.00
0	1.462	1.366	0.934	3.44	1.10
10	1.455	1.359	0.934	3.60	1.15
20	1.364	1.458	1.069	3.64	1.16
30	1.364	1.562	1.145	3.64	1.16
40	1.371	1.366	0.996	3.55	1.13
50	1.357	1.262	0.930	3.18	1.02
60	1.364	1.458	1.069	3.33	1.06
70	1.364	1.451	1.064	3.58	1.14
80	1.455	1.556	1.069	3.26	1.04
90	1.469	1.366	0.930	3.63	1.16

	Acquisition technique: 20 BPM, image binning				
	Dimension (cm)				
Phase (%)	AP	LR	Elongation^a^ (cm^3^)	Volume	Volume ratio^b^
Static	1.368	1.360	0.994	3.13	1.00
0	1.362	1.366	1.003	3.24	1.04
10	1.362	1.465	1.076	4.02	1.28
20	1.362	1.657	1.217	3.70	1.18
30	1.366	1.661	1.216	3.89	1.24
40	1.362	1.460	1.072	3.31	1.06
50	1.366	1.366	1.000	3.27	1.04
60	1.366	1.366	1.000	3.94	1.26
70	1.366	1.465	1.072	4.09	1.31
80	1.366	1.558	1.141	4.12	1.32
90	1.362	1.465	1.076	4.05	1.29

	Acquisition technique: IRR, image binning				
	Dimension (cm)				
Phase (%)	AP	LR	Elongation^a^ (cm^3^)	Volume	Volume ratio^b^
Static	1.368	1.360	0.994	3.13	1.00
0	1.370	1.360	0.993	3.47	1.11
10	1.370	1.466	1.070	3.36	1.07
20	1.364	1.355	0.993	4.28	1.37
30	1.461	1.460	0.999	3.78	1.21
40	1.370	1.366	0.997	3.95	1.26
50	1.376	1.360	0.988	3.26	1.04
60	1.460	1.461	1.001	3.64	1.16
70	1.370	1.461	1.066	3.89	1.24
80	1.370	1.455	1.062	3.58	1.14
90	1.370	1.371	1.001	3.23	1.03

	Acquisition technique: 20 BPM, projection binning				
	Dimension (cm)				
Phase (%)	AP	LR	Elongation^a^ (cm^3^)	Volume	Volume ratio^b^
Static	1.370	1.361	0.993	3.12	1.00
0	1.320	1.408	1.067	3.18	1.02
10	1.324	1.408	1.063	3.58	1.15
20	1.408	1.399	0.994	3.64	1.17
30	1.408	1.408	1.000	3.53	1.13
40	1.332	1.413	1.061	3.46	1.11
50	1.332	1.329	0.998	3.43	1.10
60	1.408	1.399	0.994	3.19	1.02
70	1.408	1.408	1.000	3.45	1.11
80	1.408	1.324	0.940	3.48	1.12
90	1.408	1.399	0.994	3.19	1.02

	Acquisition technique: IRR, projection binning				
	Dimension (cm)				
Phase (%)	AP	LR	Elongation^a^ (cm^3^)	Volume	Volume ratio^b^
Static	1.370	1.361	0.993	3.12	1.00
0	1.329	1.405	1.057	3.35	1.07
10	1.329	1.405	1.057	3.54	1.13
20	1.324	1.481	1.119	3.16	1.01
30	1.329	1.405	1.057	2.82	0.90
40	1.329	1.405	1.057	3.07	0.98
50	1.329	1.405	1.057	3.51	1.13
60	1.329	1.405	1.057	3.96	1.27
70	1.329	1.565	1.178	3.70	1.19
80	1.329	1.325	0.997	3.52	1.13
90	1.329	1.333	1.003	2.97	0.95

a “Elongation” is defined as AP / LR, where AP is the anterior–posterior dimension and LR is the left–right dimension.

b “Volume ratio” is defined as the volume of the insert in a specified phase divided by the volume of the insert as determined on the static image dataset.

BPM=breaths per minute; AP=anterior−posterior; LR=left−right; IRR=irregular breathing.

**Table 4:** Comparison of the distortion and volume of the image of the air insert in each of 10 phases of a four‐dimensional image dataset with the image of the insert in a static computed tomography dataset

	Acquisition technique: 15 BPM, image binning				
	Dimension (cm)				
Phase (%)	AP	LR	Elongation^a^ (cm^3^)	Volume	Volume ratio^b^
Static	1.368	1.365	0.998	3.09	1.00
0	1.352	1.366	1.010	3.45	1.12
10	1.352	1.359	1.005	3.82	1.24
20	1.267	1.468	1.159	3.61	1.17
30	1.274	1.476	1.159	3.88	1.26
40	1.360	1.351	0.993	3.49	1.13
50	1.360	1.359	0.999	3.42	1.11
60	1.368	1.461	1.068	3.23	1.05
70	1.360	1.454	1.069	3.68	1.19
80	1.360	1.468	1.079	3.35	1.08
90	1.368	1.373	1.004	3.65	1.18

	Acquisition technique: 20 BPM, image binning				
	Dimension (cm)				
Phase (%)	AP	LR	Elongation^a^ (cm^3^)	Volume ratio^b^	Volume
Static	1.368	1.365	0.998	3.09	1.00
0	1.370	1.366	0.997	3.50	1.13
10	1.370	1.464	1.069	4.13	1.34
20	1.276	1.558	1.221	3.83	1.24
30	1.272	1.554	1.222	4.27	1.38
40	1.370	1.366	0.997	3.19	1.03
50	1.558	1.563	1.003	4.15	1.34
60	1.272	1.464	1.151	3.89	1.26
70	1.267	1.469	1.159	4.45	1.44
80	1.267	1.464	1.155	4.11	1.33
90	1.370	1.371	1.001	4.12	1.33

	Acquisition technique: IRR, image binning				
	Dimension (cm)				
Phase (%)	AP	LR	Elongation^a^ (cm^3^)	Volume	Volume ratio^b^
Static	1.368	1.365	0.998	3.09	1.00
0	1.369	1.364	0.996	3.45	1.12
10	1.369	1.465	1.070	3.55	1.15
20	1.369	1.360	0.993	4.55	1.47
30	1.369	1.465	1.070	3.98	1.29
40	1.369	1.364	0.996	4.16	1.35
50	1.369	1.268	0.926	3.37	1.09
60	1.559	1.562	1.002	4.38	1.42
70	1.369	1.465	1.070	4.21	1.36
80	1.369	1.364	0.996	3.52	1.14
90	1.369	1.373	1.003	3.75	1.21

	Acquisition technique: 15 BPM, projection binning				
	Dimension (cm)				
Phase (%)	AP	LR	Elongation^a^ (cm^3^)	Volume	Volume ratio^b^
Static	1.360	1.270	0.934	3.27	1.00
0	1.330	1.326	0.997	3.65	1.12
10	1.330	1.408	1.059	3.76	1.15
20	1.330	1.486	1.117	3.97	1.21
30	1.330	1.486	1.117	3.89	1.19
40	1.330	1.399	1.052	3.53	1.08
50	1.330	1.408	1.059	3.48	1.06
60	1.330	1.399	1.052	3.55	1.09
70	1.330	1.486	1.117	3.87	1.18
80	1.335	1.481	1.109	3.88	1.19
90	1.330	1.331	1.001	3.50	1.07

	Acquisition technique: 20 BPM, projection binning				
	Dimension (cm)				
Phase (%)	AP	LR	Elongation^a^ (cm^3^)	Volume	Volume ratio^b^
Static	1.360	1.270	0.934	3.27	1.00
0	1.325	1.375	1.038	3.42	1.05
10	1.330	1.402	1.054	3.61	1.10
20	1.330	1.402	1.054	3.70	1.13
30	1.330	1.330	1.000	3.60	1.10
40	1.335	1.325	0.993	3.48	1.06
50	1.330	1.407	1.058	3.47	1.06
60	1.330	1.407	1.058	3.59	1.10
70	1.413	1.325	0.938	3.53	1.08
80	1.484	1.485	1.001	4.22	1.29
90	1.485	1.489	1.003	4.16	1.27

	Acquisition technique: IRR, projection binning				
	Dimension (cm)				
Phase (%)	AP	LR	Elongation^a^ (cm^3^)	Volume	Volume ratio^b^
Static	1.360	1.270	0.934	3.27	1.00
0	1.404	1.324	0.943	3.49	1.07
10	1.404	1.410	1.004	3.88	1.19
20	1.398	1.491	1.067	3.27	1.00
30	1.404	1.324	0.943	3.10	0.95
40	1328	1.324	0.001	3.11	0.95
50	1.328	1.324	0.997	3.83	1.17
60	1.328	1.405	1.058	4.00	1.22
70	1.334	1.486	1.114	4.28	1.31
80	1.405	1.405	1.000	3.90	1.19
90	1.404	1.410	1.004	3.13	0.96

a “Elongation” is defined as AP / LR, where AP is the anterior–posterior dimension and LR is the left–right dimension.

b “Volume ratio” is defined as the volume of the insert in a specified phase divided by the volume of the insert as determined on the static image dataset.

BPM=breaths per minute; AP=anterior−posterior; LR=left−right; IRR=irregular breathing.

**Table 5:** Comparison of the distortion and volume of the image of the low‐density polyethylene insert in each of 10 phases of a four‐dimensional image dataset with the image of the insert in a static computed tomography dataset

	Acquisition technique: 15 BPM, image binning				
	Dimension (cm)				
Phase (%)	AP	LR	Elongation^a^ (cm^3^)	Volume	Volume ratio^b^
Static	1.167	1.264	1.083	2.84	1.00
0	1.258	1.261	1.002	2.75	0.97
10	1.161	1.171	1.009	2.74	0.96
20	1.168	1.071	0.917	2.05	0.72
30	1.262	1.185	0.939	2.36	0.83
40	1.268	1.164	0.918	2.53	0.89
50	1.268	1.273	1.004	2.59	0.91
60	1.262	1.180	0.935	2.24	0.79
70	1.268	1.159	0.914	2.18	0.77
80	1.174	1.066	0.908	2.16	0.76
90	1.168	1.169	1.001	2.78	0.98

	Acquisition technique: 20 BPM, image binning				
	Dimension (cm)				
Phase (%)	AP	LR	Elongation^a^ (cm^3^)	Volume	Volume ratio^b^
Static	1.167	1.264	1.083	2.84	1.00
0	1.170	1.267	1.083	2.57	0.90
10	1.173	1.166	0.994	2.23	0.79
20	1.173	1.271	1.084	1.60	0.56
30	1.173	1.069	0.911	2.29	0.81
40	1.271	1.267	0.997	2.25	0.79
50	1.271	1.267	0.997	2.63	0.93
60	1.270	1.170	0.921	2.82	0.99
70	1.173	1.076	0.917	2.06	0.73
80	1.173	1.170	0.997	2.31	0.81
90	1.170	1.173	1.003	2.17	0.76

	Acquisition technique: IRR, image binning				
	Dimension (cm)				
Phase (%)	AP	LR	Elongation^a^ (cm^3^)	Volume	Volume ratio^b^
Static	1.167	1.264	1.083	2.84	1.00
0	1.172	1.264	1.078	2.60	0.92
10	1.172	1.271	1.084	2.60	0.92
20	1.172	1.267	1.081	2.78	0.98
30	1.169	1.176	1.006	2.77	0.98
40	1.176	1.165	0.991	2.84	1.00
50	1.172	1.267	1.081	2.55	0.90
60	1.176	1.264	1.075	2.59	0.91
70	1.172	1.169	0.997	2.64	0.93
80	1.172	1.172	1.000	2.33	0.82
90	1.275	1.169	0.917	2.59	0.91

	Acquisition technique: 15 BPM, projection binning				
	Dimension (cm)				
Phase (%)	AP	LR	Elongation^a^ (cm^3^)	Volume	Volume ratio^b^
Static	1.171	1.169	0.998	2.57	1.00
0	1.248	1.240	0.994	2.91	1.13
10	1.248	1.248	1.000	2.55	0.99
20	1.248	1.166	0.934	2.77	1.08
30	1.248	1.251	1.002	2.80	1.09
40	1.248	1.244	0.997	2.84	1.11
50	1.248	1.251	1.002	2.88	1.12
60	1.248	1.248	1.000	2.55	0.99
70	1.248	1.251	1.002	2.44	0.95
80	1.248	1.246	0.998	2.94	1.14
90	1.248	1.246	0.998	2.61	1.02

	Acquisition technique: 20 BPM, projection binning				
	Dimension (cm)				
Phase (%)	AP	LR	Elongation^a^ (cm^3^)	Volume	Volume ratio^b^
Static	1.171	1.169	0.998	2.57	1.00
0	1.255	1.254	0.999	2.83	1.10
10	1.255	1.260	1.004	2.88	1.12
20	1.255	1.173	0.935	2.79	1.09
30	1.255	1.248	0.994	2.61	1.02
40	1.243	1.167	0.939	3.01	1.17
50	1.243	1.248	1.004	2.94	1.14
60	1.174	1.161	0.989	2.70	1.05
70	1.249	1.173	0.939	2.99	1.16
80	1.254	1.255	1.001	2.59	1.01
90	1.255	1.242	0.990	1.91	0.74

	Acquisition technique: IRR, projection binning				
	Dimension (cm)				
Phase (%)	AP	LR	Elongation^a^ (cm^3^)	Volume	Volume ratio^b^
Static	1.171	1.169	0.998	2.57	1.00
0	1.172	1.244	1.061	2.64	1.03
10	1.172	1.173	1.001	2.30	0.89
20	1.172	1.244	1.061	2.13	0.83
30	1.172	1.249	1.066	2.70	1.05
40	1.248	1.254	1.005	2.54	0.99

	Acquisition technique: IRR, projection binning				
	Dimension (cm)				
Phase (%)	AP	LR	Elongation^a^ (cm^3^)	Volume	Volume ratio^b^
50	1.272	1.244	0.978	2.84	1.11
60	1.172	1.249	1.066	3.11	1.21
70	1.172	1.249	1.066	3.09	1.20
80	1.172	1.249	1.066	2.97	1.16
90	1.172	1.249	1.066	2.60	1.01

a “Elongation” is defined as AP / LR, where AP is the anterior‐posterior dimension and LR is the left‐right dimension.

b “Volume ratio” is defined as the volume of the insert in a specified phase divided by the volume of the insert as determined on the static image dataset.

BPM=breaths per minute; AP=anterior−posterior; LR=left−right; IRR=irregular breathing.

As indicated in Tables 3 – 5, the calculated volumes of the CT inserts on the phases of the 4D image datasets were typically comparable with those on the static datasets. The largest deviations occurred at the phases that corresponded to the greatest motion of the platform. Examination of the outlines on the CT image datasets indicate that, at these phases, images of the inserts appeared on additional transverse planes at either end of the image of the inserts. The additional planes in which the contours occurred are artifacts of the residual motion. The decision to include these planes was based solely on the CT numbers used as the threshold; a different threshold value might have resulted in these planes not being included in the volume of the insert.

### C. Accuracy of voxel values

**Figure 5:**
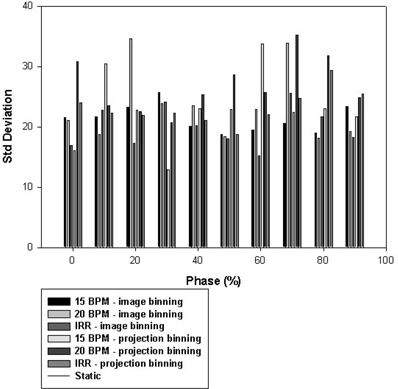
Plot of the standard deviation (SD) of the computed tomography (CT) number against phase for the Teflon insert for the various motion configurations in the study.

Tables 6‐8 show the statistics for the CT voxel values of each of the three sampled Catphan phantom cylindrical target inserts. The tables compare the mean voxel values and standard deviations of the voxel values of the inserts on the phases of the 4D CT image dataset, plus the values on the static dataset. The mean voxel values for the inserts in the 4D images did not differ from the values on the static images, but the standard deviations of the voxel values exhibited larger differences, indicating noisier images. Regions of interest in the images of the inserts exhibited greater standard deviations in the phases that corresponded to greater motion of the platform, as shown in Fig. 5, which plots the standard deviations of the voxel values for the LDPE insert against phase. At the 0% and 50% phases, corresponding to end inspiration and end expiration respectively, the displacement of the phantom is the smallest. These phases also correspond to the least amount of noise in the CT images. At the 20%−30% and 70%−80% phases, for which displacement of the phantom is greatest, the largest amount of noise is observed.

**Table 6:** Mean computed tomography (CT) voxel value and standard deviation (SD) of the CT voxel values for the image of the Teflon insert in each of the 10 phases of a four‐dimensional image dataset and in a static CT dataset

		Acquisition technique: 15 BPM, image binning		
Phase (%)	Mm	Max	Mean	SD
Static	1930	1961	1947	6.83
0	1833	1942	1897	21.53
10	1843	1963	1895	21.63
20	1852	1979	1918	23.19
30	1838	1970	1918	25.73
40	1871	1958	1917	20.11
50	1858	1949	1903	18.68
60	1857	1940	1897	19.47
70	1829	1919	1877	20.56
80	1840	1948	1911	18.97
90	1849	1953	1898	23.38

		Acquisition technique: 20 BPM, image binning		
Phase (%)	Mm	Max	Mean	SD
Static	1930	1961	1947	6.83
0	1848	1949	1894	21.02
10	1858	1953	1902	18.77
20	1719	1938	1884	34.57

		Acquisition technique: 20 BPM, image binning		
Phase (%)	Mm	Max	Mean	SD
30	1806	1960	1914	23.80
40	1844	1970	1912	23.50
50	1880	1974	1911	18.41
60	1854	1955	1911	22.89
70	1734	1942	1890	33.82
80	1866	1948	1903	18.11
90	1858	1938	1898	19.17

		Acquisition technique: IRR, image binning		
Phase (%)	Mm	Max	Mean	SD
Static	1930	1961	1947	6.83
0	1864	1944	1902	16.94
10	1855	1943	1899	22.74
20	1854	1936	1892	17.27
30	1857	1972	1908	24.09
40	1873	1970	1919	20.18
50	1848	1934	1896	18.03
60	1857	1934	1895	15.14
70	1848	1963	1904	25.61
80	1870	1977	1912	21.65
90	1846	1935	1894	18.26

		Acquisition technique: 15 BPM, projection binning		
Phase (%)	Mm	Max	Mean	SD
Static	1911	1940	1947	5.25
0	1869	1944	1902	16.10
10	1823	1963	1895	30.38
20	1820	1937	1900	22.72
30	1877	1930	1900	12.87
40	1846	1954	1898	23.01
50	1840	1951	1898	22.91
60	1819	1991	1905	33.77
70	1839	1940	1901	22.42
80	1818	1959	1902	22.95
90	1840	1943	1898	21.67

		Acquisition technique: 20 BPM, projection binning		
Phase (%)	Mm	Max	Mean	SD
Static	1911	1940	1947	5.25
0	1820	1980	1901	30.80
10	1847	1952	1895	23.52
20	1854	1949	1899	22.45
30	1846	1946	1896	20.69
40	1855	1984	1906	25.35
50	1855	1952	1900	19.83
60	1813	1954	1892	25.70
70	1806	1998	1899	35.18
80	1832	1977	1903	31.74
90	1847	1966	1900	24.79

		Acquisition technique: IRR, projection binning		
Phase (%)	Mm	Max	Mean	SD
Static	1911	1940	1947	5.25
0	1838	1957	1905	24.03
10	1850	1965	1900	22.21
20	1819	1952	1902	21.92
30	1835	1949	1894	22.22

		Acquisition technique: IRR, projection binning		
Phase (%)	Mm	Max	Mean	SD
40	1860	1950	1899	21.04
50	1854	1952	1893	18.68
60	1845	1949	1897	22.08
70	1768	1938	1897	24.75
80	1822	1980	1900	29.33
90	1714	1836	1776	25.45

BPM=breaths per minute; IRR=irregular breathing.

**Table 7:** Mean computed tomography (CT) voxel value and standard deviation (SD) of the CT voxel values for the image of the air insert in each of the 10 phases of a four‐dimensional image dataset and in a static CT dataset

		Acquisition technique: 15 BPM, image binning		
Phase (%)	Mm	Max	Mean	SD
Static	0	21	9	5.05
0	12	96	53	16.25
10	24	90	54	13.51
20	16	94	40	15.55
30	0	108	32	19.55
40	21	79	49	13.20
50	0	77	32	15.24
60	6	83	49	14.53
70	3	75	38	13.60
80	24	148	58	20.43
90	4	66	40	13.72

		Acquisition technique: 20 BPM, image binning		
Phase (%)	Mm	Max	Mean	SD
Static	0	21	9	5.05
0	11	85	40	18.69
10	8	85	40	17.18
20	0	177	38	33.73
30	23	312	66	48.31
40	17	94	53	14.98
50	0	61	28	12.29
60	28	104	58	15.21
70	21	93	55	15.49
80	10	90	48	15.54
90	0	150	34	23.15

		Acquisition technique: IRR, image binning		
Phase (%)	Mm	Max	Mean	SD
Static	0	21	9	5.05
0	14	86	50	17.08
10	16	86	52	16.08
20	48	135	83	16.73
30	4	61	36	12.86
40	2	61	29	13.93
50	15	84	46	15.16
60	25	89	60	13.76
70	0	88	54	16.58
80	15	85	51	16.13
90	24	97	53	17.25

		Acquisition technique: 15 BPM, projection binning		
Phase (%)	Mm	Max	Mean	SD
Static	17	52	33	7.1
0	6	74	45	14.88
10	6	83	44	15.57
20	3	88	43	16.90
30	6	113	48	19.98
40	6	79	38	14.12
50	1	71	38	15.53
60	98	185	136	18.61
70	21	94	48	14.09
80	17	88	43	11.05
90	0	96	42	20.76

		Acquisition technique: 20 BPM, projection binning		
Phase (%)	Mm	Max	Mean	SD
Static	17	52	33	7.1
0	0	78	38	18.53
10	0	110	49	25.59
20	0	82	43	13.95
30	0	106	39	23.11
40	5	119	50	21.13
50	1	94	42	21.46
60	33	116	69	17.21
70	9	94	45	18.96
80	5	100	42	20.86
90	0	117	46	25.21

		Acquisition technique: IRR, projection binning		
Phase (%)	Mm	Max	Mean	SD
Static	17	52	33	7.1
0	0	86	39	20.41
10	6	86	43	15.86
20	0	94	44	17.55
30	0	92	39	20.13
40	0	100	45	18.20
50	5	86	46	17.64
60	12	74	40	11.97
70	0	132	39	18.19
80	0	98	42	20.26
90	4	85	42	14.52

BPM=breaths per minute; IRR=irregular breathing.

**Table 8:** Mean computed tomography (CT) voxel value and standard deviation (SD) of the CT voxel values for the image of the low‐density polyethylene insert in each of the 10 phases of a four‐dimensional image dataset and in a static CT dataset

		Acquisition technique: 15 BPM, image binning		
Phase (%)	Mm	Max	Mean	SD
Static	896	931	912	7.82
0	875	959	919	17.80
10	871	977	920	20.20
20	879	962	916	19.02
30	871	943	911	17.56
40	883	953	920	17.03
50	884	958	918	18.93
60	873	949	917	15.84

		Acquisition technique: 15 BPM, image binning		
Phase (%)	Mm	Max	Mean	SD
70	847	955	908	22.57
80	873	971	922	19.11
90	879	958	921	18.50

		Acquisition technique: 20 BPM, image binning		
Phase (%)	Mm	Max	Mean	SD
Static	896	931	912	7.82
0	871	971	924	20.01
10	881	970	925	19.77
20	865	959	906	20.39
30	879	971	923	20.73
40	857	965	916	20.24
50	886	973	920	17.12
60	877	980	920	21.54
70	855	951	919	20.15
80	880	995	922	23.53
90	868	963	916	21.34

		Acquisition technique: IRR, image binning		
Phase (%)	Mm	Max	Mean	SD
Static	896	931	912	7.82
0	866	949	916	16.92
10	882	969	919	17.41
20	870	961	917	18.74
30	873	957	919	21.25
40	857	955	914	20.68
50	869	941	912	14.86
60	878	969	922	18.14
70	893	964	924	16.08
80	869	954	924	14.95
90	859	964	922	20.59

		Acquisition technique: 15 BPM, projection binning		
Phase (%)	Mm	Max	Mean	SD
Static	906	928	917	5.01
0	873	948	911	14.22
10	859	973	915	25.27
20	872	946	914	17.24
30	876	949	911	14.96
40	880	969	914	14.50
50	874	955	912	16.30
60	847	960	916	24.17
70	884	959	915	14.87
80	870	952	914	15.17
90	835	959	913	23.96

		Acquisition technique: 20 BPM, projection binning		
Phase (%)	Mm	Max	Mean	SD
Static	906	928	917	5.01
0	864	958	912	18.54
10	846	964	908	23.45
20	869	970	916	24.47

		Acquisition technique: 20 BPM, projection binning		
Phase (%)	Mm	Max	Mean	SD
30	862	954	912	19.28
40	853	982	915	25.94
50	864	962	918	21.57
60	874	978	908	19.80
70	852	983	917	26.94
80	860	977	917	21.82
90	872	986	915	21.70

		Acquisition technique: IRR, projection binning		
Phase (%)	Mm	Max	Mean	SD
Static	906	928	917	5.01
0	862	964	915	24.26
10	877	972	915	19.51
20	876	958	918	17.25
30	850	972	908	27.24
40	868	954	912	16.29
50	851	963	912	22.55
60	867	934	904	16.01
70	874	955	913	17.79
80	859	959	906	21.11
90	836	986	913	26.10

BPM=breaths per minute; IRR=irregular breathing.

## IV. DISCUSSION

In 4D CT imaging, respiratory motion has been shown to generate artifacts resulting from interplay between acquisition of CT data and object motion.^(6)^ Artifacts may be reduced by careful selection of gantry rotation speed and table translation speed,^(7)^ thus resulting in improved image quality. The present study has demonstrated several parameters that can be used to quantitatively assess the quality of 4D CT image generation and how those parameters can be applied to images generated using two different approaches to 4D CT image reconstruction. As a result, we found that, unless artifacts occur because of irregular motion, the geometric reproducibility of anatomic structures on phases from 4D CT images appears to be within the uncertainty of contouring the anatomic structures and, hence, are acceptable for treatment planning purposes. Problems may exist for threshold‐based autocontouring, primarily because of partial‐volume effects. It appears that, when an autocontouring algorithm is used to assist in contouring anatomy in multiple phases of a 4D study, it is necessary to introduce correlation among contours representing the same region of interest at various phases. Deformable model segmentation, in which an anatomic structure or target volume is delineated in one phase and is then transferred to other phases, may have a role in this application.^(^ ^8^ ^,^ ^9^ ^)^

From one phase to the next, CT voxel values maintained their integrity, but 4D images tended to suffer from residual motion artifacts. It may be possible to reduce this spread of voxel values on the 4D CT images, but such a reduction could require an increased dose to the patient and may not necessarily be desirable. Some studies have indicated that it may be possible to reduce variation on a single phase of a 4D CT image set by retrospectively deforming all other phases to the desired phase and then combining images,^(10)^ or by using displacement binning techniques.^(^ ^11^ ^,^ ^12^ ^)^

Our findings are similar to those of Wink et al.^(7)^ who performed similar studies with a CT scanner that used the PB technique.^(3)^ Their gantry rotation speeds, which resulted in cycle times of 0.5 – 1.0 s, were slower than those used in the present study, and their pitches of 0.1 – 0.45 were much greater. We concur with Wink et al., who indicated that the fastest possible gantry rotation speed should be used to minimize the effects of motion. Table travel during one gantry rotation that is greater than the width of the detector may result in spatial gaps in the acquisition of projection data. To ensure that the table travel during one respiratory cycle is less than the width of the detector during use of the PB approach, the pitch must be kept small, with the maximum value given by the equation

$$\text{pitch}\leq \frac{\text{gantry}\;\text{rotation}\;\text{time}\;(\sec)\;\times \;\text{respiratory}\;\text{rate}\;(\min^{-1})}{60\;\sec/\min}$$

For a gantry rotation time of 0.4 s, which is the fastest speed available on the CT scanner that supports PB, and a respiratory rate of 15 breaths per minute, the pitch must be kept below 0.1. A respiratory rate of 15 breaths per minute is considered to be a relatively rapid respiratory cycle; many patients exhibit respiratory rates of 10 – 12 breaths per minute; consequently, pitches not exceeding 0.06 – 0.08 are required, with even lower pitches are desirable. We further concur with Wink et al., and with other authors,^(^ ^4^ ^,^ ^7^ ^,^ ^13^ ^,^ ^14^ ^)^ who indicated that, with proper selection of image acquisition parameters, image distortion under 4D CT should not be significant and the technique should provide better image definition than conventional CT imaging.^(15)^

Some inaccuracy was observed in phase acquisition using the IB approach.^(1)^ Accuracy can be improved by reducing the time between image reconstructions. The tradeoff in this interval reduction is that more CT images are generated, which should not be a storage issue if only the images used in the 4D dataset are kept. These images are reconstructed at no cost to the patient in terms of dose. In earlier versions of 4D reconstruction software, we were limited to the generation of 1500 images, a limit that was easily reached, resulting in compromises between the interval between image reconstructions and cine duration of image acquisition at each indexed table position. With newer software that increases the limit to 3000 images, these compromises may no longer be necessary.

## V. CONCLUSIONS

We demonstrated that a phantom of known geometry resting on a platform with known motion can be used to quantitatively assess 4D CT imaging for applications in radiation oncology treatment planning. Specific assessments that can be made include accuracy of phase reconstruction, image distortion, and voxel value stability. These assessments may be useful in determining optimal sets of image acquisition parameters for 4D CT image acquisition.

Again, it should be noted that data for the two different methods of CT image acquisition should not be directly compared, because optimization of image acquisition parameters has not yet been conducted. Consequently, the particular data presented in this paper should not be used to draw any conclusions about the relative merits of the two different approaches to 4D CT reconstruction.

## ACKNOWLEDGMENTS

This work was supported in part by a Sponsored Research Agreement with Philips Medical Systems. The authors thank Drs. Geoffrey Ibbott and David Followill of the Radiological Physics Center for loan of the RPC motion platform.
